# Does food insecurity compromise diet quality among Finnish private sector service workers?

**DOI:** 10.1017/S1368980024002386

**Published:** 2024-11-22

**Authors:** Roosa Joutsi, Hanna M. Walsh, Elviira Lehto, Tiina Saari, Ossi Rahkonen, Jaakko Nevalainen, Maijaliisa Erkkola, Jelena Meinilä

**Affiliations:** 1 Department of Food and Nutrition, University of Helsinki, Helsinki, Finland; 2 Work Research Centre, Faculty of Social Sciences, Tampere University, Tampere, Finland; 3 Department of Public Health, University of Helsinki, Helsinki, Finland; 4 Health Sciences, Faculty of Social Sciences, Tampere University, Tampere, Finland

**Keywords:** Food insecurity, Health inequality, Low socio-economic status, Nutrition

## Abstract

**Objective:**

To investigate the association between food insecurity (FI) and diet quality in private sector service workers.

**Design:**

Data were collected via electronic questionnaires (2019) and the national register data (2018–2019). FI was measured using the Household Food Insecurity Access Scale (HFIAS) and diet quality using an FFQ and a modified Healthy Food Intake Index (mHFII). The associations between HFIAS and mHFII were studied using ANOVA and ordinal regression analysis.

**Setting:**

Cross-sectional survey and register data for all municipalities in Finland in 2018–2019.

**Participants:**

Individuals (*n* 6435) belonging to the Finnish Service Union United. The members are predominantly women and work mainly in retail trade, tourism, restaurant and leisure services, property maintenance and security services.

**Results:**

Overall diet quality, measured by mHFII, was significantly lower in those experiencing severe FI than in those who were food secure (8·0 *v*. 9·1). Additionally, those with severe FI were less likely to have higher (more optimal) scores in sugar-sweetened beverages (OR: 0·67), fibre-rich grains (OR: 0·79), vegetables (OR: 0·54), fruits and berries (OR: 0·61), vegetable oil (OR: 0·80), fish (OR: 0·65), milk (OR: 0·89) and nuts and seeds (OR: 0·66) than food-secure participants. Severe FI was associated with higher odds for less frequent consumption of red and processed meat (OR: 1·15, a higher score represents less frequent consumption).

**Conclusions:**

Severe FI was linked to both lower overall diet quality and suboptimal consumption of several food groups. Individuals experiencing severe FI may be predisposed to accumulating dietary risk factors for chronic diseases.

According to the World Food Summit (1996) definition, ‘food security exists when all people, at all times, have physical and economic access to sufficient, safe, and nutritious food that meets their dietary needs and food preferences for an active and healthy life’. Food insecurity is defined as a lack of availability or access to food or a lack of capacity to utilise food to provide an adequate diet. Food insecurity has been linked to adverse health outcomes, such as cardiometabolic conditions^(1)^ and diabetes^(2)^, as well as higher mortality than in food-secure populations^(3)^.

Food insecurity has been commonly researched by the Organisation for Economic Co-operation and Development in low- to middle-income countries, and food insecurity data in the USA have been published by the US Department of Agriculture since 1995. However, it is only within the past decade that rising food insecurity in Europe has gained more attention^(4–7)^. Limited data are available in Finland. According to the FAO of the UN’s report, the prevalence of severe food insecurity in Finland was 2 %, while in 2017, the prevalence of moderate or severe food insecurity was 8·3 %^(8)^. A Finnish study from 2001 of a nationally representative sample of 25–64-year-olds^(9)^ noted that 9 % reported fears of running out of food due to economic problems, 11 % had experiences of running out of money to buy food and 3 % had had too little food due to lack of money. In a previous study on a group of private sector service workers belonging to the Finnish Service Union United (PAM), 36 % of participants were severely food insecure, and 29 % were mildly or moderately food insecure^(10)^. Furthermore, participants reported worse self-perceived health than the population average^(11)^. In Finland, the union membership rate in the private service sector was 48 % in 2017^(12)^.

The negative associations between food insecurity and health have been hypothesised to not only be direct but also mediated by an unhealthy diet^(13)^, as it is an established risk factor for many chronic diseases^(14)^. Several studies in Western societies have linked food insecurity to overall lower diet quality and particularly lower consumption of fruits and vegetables^(13,15–17)^. Up to the last decade, most studies have been conducted in the USA^(13,18)^, but reports from Europe have emerged in recent years^(15,16,19,20)^. To date, only one study has been carried out in the Nordic countries^(15)^. In this Danish study, Lund *et al.* found that after adjustment for sociodemographic factors, adults from low or very low food-secure households had a higher probability of having an unhealthy diet, as measured by the Danish Dietary Quality Score, and food insecurity was associated with lower intakes of fruits, vegetables and fish. It is important to investigate these associations in Nordic welfare countries since dietary patterns, social safety nets, food availability and price policies, among others, differ from the USA and other European countries.

In high-income countries, diet quality tends to follow a socio-economic gradient where higher quality diets are associated with higher socio-economic status^(21)^. According to a Finnish report, one of the socio-economic factors linked to low diet quality was occupational class; blue-collar workers had on average lower-quality diets than white-collar workers^(22)^. Thus, it is important to identify the most vulnerable groups for whom the dietary risk factors could accumulate. Evidence shows that people in lower socio-economic groups, including lower occupational class, are at higher risk of both lower diet quality and higher food insecurity^(15,21,23–25)^. It remains unclear whether food insecurity exacerbates the poor diet quality observed in lower socio-economic groups such as Finnish private sector workers in low-salary positions.

The aim of this study was to investigate the association between food insecurity and diet quality in private sector service workers who were members of PAM and to analyse differences in consumption of selected food groups across the different levels of food insecurity.

## Methods

### Study design and participants

Data were collected via two online surveys in collaboration with Service Union United PAM, the trade union for private sector service workers. Most PAM members work in retail trade, tourism, restaurant and leisure services, property maintenance services (including cleaning) or security services. PAM has 190 000 members, 71 % of whom are women^(26)^.

In April–May 2019, the PAMEL study survey was sent to all Finnish-speaking employed, unemployed and retired PAM members who had provided their email addresses in the PAM member register, excluding student members (*n* 111 850). The number of individuals receiving or reading the email is unknown. The study survey included questions on food consumption frequency, food insecurity and sociodemographic characteristics. Data on the employment industry were obtained from the annual PAM survey sent in May–June 2019.

Participants were asked for permission to link their survey answers to national register data provided by Statistics Finland for the years 2018–2019. Data obtained from Statistics Finland for 2019 included sex, year of birth, municipality type and individual income for 2018. Of the 6435 participants who answered the PAMEL study survey, national register data were available for 6431 members in 2018 and for 6421 members in 2019.

### Measures

*Food insecurity* was measured with a slightly modified Household Food Insecurity Access Scale (HFIAS), originally developed by Coates *et al.*^(27)^. Although other food insecurity questionnaires are available (e.g. Food Insecurity Experience Scale by the FAO of the UN)^(28)^, we chose to use the modified HFIAS because it was previously validated in the same study population^(10)^. The tool was translated into Finnish and modified to inquire about an individual’s food insecurity experience rather than the entire household’s, as described in Walsh *et al.*^(10)^.

The HFIAS questionnaire includes nine questions on how often participants have experienced issues related to worry about having enough food or having to limit the food quality or quantity for financial reasons during the past 30 d. Based on their responses, participants were categorised as food secure or mildly, moderately or severely food insecure^(27)^. In the previous study among the same sample of service workers, the HFIAS tool demonstrated acceptable construct and criterion validity^(11)^.

*Food consumption* was measured with an FFQ inquiring about the frequency of consumption of different food items over the last month. The FFQ was designed to measure the whole diet of participants and was based on an FFQ that has previously shown acceptable validity when ranking food group consumption compared with food records in Finnish children^(29)^. The FFQ was modified for adults: Some food items were combined into broader food groups (cheese instead of low-fat and high-fat cheese, yogurt instead of natural and flavoured yogurt, breakfast cereals instead of sugar-sweetened breakfast cereals and whole-grain breakfast cereals, sweet pastry instead of biscuits and cakes), and some food items were added (oils, margarines, oil-based salad dressings, coffee, tea, bottled water, wine, beer, cider, alcohol-free beer and cider and spirits), and finally, dried fruits and berries and flavoured nuts were removed. Because of these changes, the number of food items in the final modified FFQ was fifty-two food items (instead of forty-seven in the original). The FFQ was modified to concern the previous month instead of the previous week as in the original FFQ, with the seven response options ranging accordingly from ‘not at all’ to ‘more than once a day’. The frequency options were converted to weekly values as follows (converted values in parentheses): not at all (0 times per week), less than once a month (0·12 times per week), 1–3 d per month (0·47 times per week), 1–2 d per week (1·5 times per week), 3–5 d per week (4 times per week), daily or almost daily (6 times per week) and more than once a day (8 times per week). Portion sizes were not included in the form.

*Diet quality* was measured with a modified version of the Healthy Food Intake Index (HFII), developed and validated by Meinilä *et al.*^(30)^. The HFII components reflect the food-based dietary guidelines of the Nordic Nutrition Recommendations^(31)^. In a validation study, higher HFII scores reflected nutrient intake closer to the recommendations, and scores were higher in those who had higher educational attainment, were more physically active, had lower BMI or were non-smokers^(30)^. Detailed FFQ data allowed us to incorporate processed and red meat as well as nuts and seeds in the original HFII to create a modified HFII (mHFII). Certain cut-off points had to be altered since our FFQ response options differed from the original.

A detailed description of the foods included in the different food groups of the index and the frequency cut-off points can be found in Table 1. In brief, items of fast food and low-fat cheese were removed as those were not included in the FFQ, and red and processed meat and nuts and seeds were added because the Finnish and Nordic Nutrition Recommendations include a recommendation for both food groups^(31,32)^. The modified index comprised eleven food groups for which points between 0 and 2 or between 0 and 1 were awarded based on the frequency of consumption and weighting. The maximum score was higher (2 points *v*. 1 point) for food groups considered to have relatively more importance in the Finnish diet. The total index score ranged from 0 to 18, with a higher score indicating more optimal consumption.

**Table 1.** Description of modified healthy food intake index (mHFII)

*Reasoning for higher score*	Food group	Foods included from the FFQ	Score	Intake frequency
*Better quality*	Fat spread	Butter, margarine	2	Margarine (> 60 % fat) > 1–3 ×/month (and others less than that)
			1	Margarine (< 60 % fat) > 1–3 ×/month OR more than one spread > 1–3 ×/month
			0	Butter > 1–3 ×/month (and others less than that)
	Milk	All milks, sour milk, plant milk	2	Low-fat milk and sour milk > 1–3 ×/month (and others less than that)
			1	A mixture of low and full-fat milk and/or plant-based milk > 1–3 ×/month
			0	Full-fat milk > 1–3 ×/month or no milk (=all milk intake < 1–3 ×/month)
*Less frequent consumption*	Snacks	Sweets, chocolate, sweet pastries, crisps, popcorn, salted nuts, ice cream	2	< 3 ×/week
			1	3–6 ×/week
			0	> 6 ×/week
	Sugar-sweetened beverages	Sugar-sweetened soft drink, juice	1	≤ 1–3 ×/month
			0	> 1–3 ×/month
	Processed and red meat	Red meat, cold cuts, sausages, frankfurters	2	≤ 3 ×/week
			1	4–5 ×/week
			0	> 5 ×/week
*More frequent consumption*	Fibre-rich grains	Dark rice and pasta, rye bread, crispy bread, white whole-grain bread, porridge	2	≥ 12 ×/week
			1	6–11 ×/week
			0	< 6 ×/week
	Vegetable oil	Vegetable oil in food preparation, oil-based salad dressing	1	≥ 1–2 ×/week
			0	< 1–2 ×/week
	Vegetables	Fresh, cooked and canned vegetables, legumes, pulses	2	> 12 ×/week
			1	6–12 ×/week
			0	< 6 ×/week
	Fruits and berries	Fresh, canned and frozen fruits, fresh and frozen berries	1	≥ 6 ×/week
			0	<6 ×/week
	Fish	Fish foods and products	2	≥1 ×/week
			0	<1 ×/week
	Nuts and seeds	Non-salted nuts, almonds, seeds	1	≥ 3–5 ×/week
			0	< 3–5 ×/week
Total			18	

### Statistical analyses

The normality of the variables was assessed through visual inspection separately in each HFII and food insecurity category. The association between food insecurity levels and modified mHFII score was first tested with one-way ANOVA, followed by estimation of pairwise differences to the reference level (food secure). Associations between sociodemographic variables and food insecurity were described by Walsh *et al.*^(10)^.

Multi-way ANOVA was then used to refine the estimates after adjustment for confounding variables. The confounding variables were identified using a combination of hypothesis-driven and data-driven methods. First, potential confounders were identified from previous studies^(33–36)^. These comprised age, sex, highest education, marital status, household size, number of children in the household, housing type (e.g. owner-occupied housing, rented municipal housing), municipality type (urban, semi-rural, rural), employment status and income.

Second, key confounders were defined as those significantly associated with both food insecurity and mHFII score, influencing the estimates of food insecurity levels, and doing so persistently and with little correlation with the other key confounders. Following these criteria, key confounders in the multi-way ANOVA model were age, sex and highest education. The associations of these variables and the mean of the mHFII scores were tested with one-way ANOVA.

Furthermore, the associations between food insecurity and individual food group scores were examined with ordinal regression analysis using a proportional odds model. Because our analyses revealed that mHFII scores differed significantly only between those who experienced severe food insecurity and those who were food secure, we compared only these two groups. Analyses were conducted for each of the eleven food groups, where the outcome variable was the food group score (either two or three score categories), and the explanatory variable was food insecurity level (severe food insecurity/food secure). The ordinal regression models were adjusted for the key confounders.

Missing data were excluded from the analyses. The level of statistical significance used was set at 0·05. To control multiplicity, pairwise comparisons were Bonferroni-corrected. All statistical analyses were performed using the SPSS statistical software package, version 27 (SPSS Inc.).

## Results

### Participants

Sample characteristics and the associations of sociodemographic variables with mHFII score are presented in Table 2. Most of the participants were women (80 %), and the largest age groups were 30–44-year-olds (34 %) and 45–59-year-olds (37 %). Most (72 %) of the participants reported their highest education as upper secondary school or vocational education.

**Table 2.** Sample characteristics and associations of sociodemographic variables with the mean of the modified healthy food intake index (mHFII) scores, in Finnish private sector service workers (*n* 6435) in 2019

	*n*	%	mHFII, mean	sd	*P* *
Total sample	6435	100	8·6	2·9	
Age, years					<0·001
17–29	987	15	8·0	2·8	
30–44	2214	34	8·1	2·8	
45–59	2381	37	8·8	2·8	
60+	839	13	9·9	2·8	
Missing data	14	0·2	9·2	1·7	
Sex					<0·001
Female	5120	80	8·7	2·9	
Male	1301	20	8·3	2·8	
Missing data	14	0·2	9·2	1·7	
Highest education					<0·001
Obligatory education or less	708	11	8·7	2·9	
Upper secondary school or vocational	4655	72	8·5	2·8	
Undergraduate	964	15	9·0	2·9	
Postgraduate	104	1·6	9·3	2·6	
Missing data	4	0·1	9·0	2·2	

* One-way ANOVA.

Mean mHFII was consistently higher in older age groups; the oldest age group had the highest mHFII score, with a score of 1·9 points higher than the youngest age group. Females had a 0·4-point higher score than males. Participants with postgraduate level education had the highest score, with a 0·8-point difference from the group with the lowest score, which was the participants with upper secondary or vocational education. There were no differences in HFII between different job industry sectors (retail, hospitality, property maintenance and others; data not shown).

### Diet quality and food insecurity

In the unadjusted model, diet quality, as measured by mean mHFII scores, was significantly lower in all three levels of food insecurity than in the food-secure level, and this difference was the most prominent among those with severe food insecurity (Table 3). The severe food insecurity level had a 1·1 (95 % CI –1·3, –0·95) point lower mean mHFII score than the food-secure level. Differences between mild and moderate food insecurity levels were approximately one-third of this difference.

**Table 3.** Mean difference in diet quality measured by modified healthy food intake index (mHFII) at different food insecurity levels, with food-secure participants as a reference group, in Finnish private sector service workers (*n* 6435) in 2019

Food insecurity level	*n*	mHFII		Difference from reference category	95 % CI	*P*
		Mean	sd			
Unadjusted model	6435					<0·001
Food secure	2280	9·1	2·9	–0		
Mildly food insecure	743	8·7	2·9	–0·4	–0·6, –0·1	
Moderately food insecure	1113	8·8	2·8	–0·3	–0·5, –0·1	
Severely food insecure	2299	8·0	2·8	–1·1	–1·3, –0·95	
Adjusted model*	6417†					<0·001
Food secure	2274			+0		
Mildly food insecure	742			–0·2	–0·5, –0·01	
Moderately food insecure	1111			–0·2	–0·4, +0·05	
Severely food insecure	2290			–0·8	–1·0, –0·6	

* Adjusted for age, sex and highest education.

† Data on age, sex and/or highest education missing for eighteen participants.

After adjusting for age, sex and education level, differences in diet quality remained significant only between severe food insecurity and food-secure levels. The mHFII score was on average 0·8 points (95 % CI –1·0, –0·6) lower in the severely food-insecure group than in the food-secure group.

### Food groups and food insecurity

**Figure 1.**
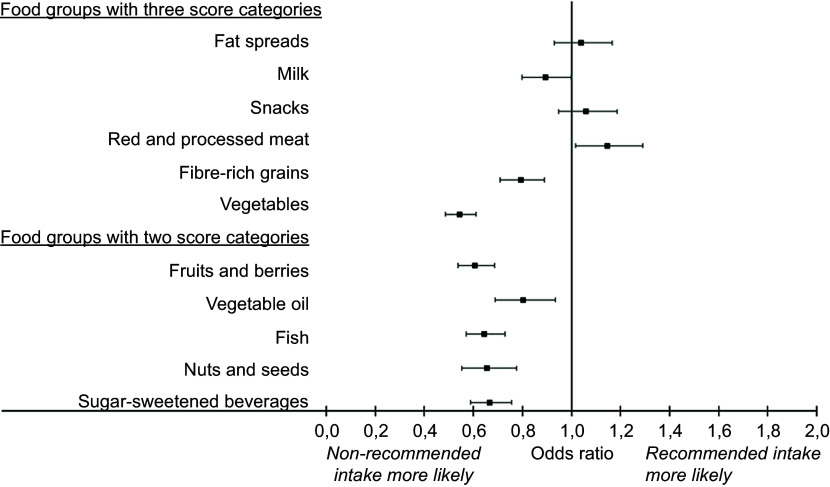
Adjusted likelihood of severely food-insecure participants achieving higher food group scores relative to food-secure participants (represented by a score of 1·0 on the scale) in Finnish private sector service workers (*n* 4564–4579) in 2019.

Compared with food-secure participants, those with severe food insecurity had nearly twofold lower odds for high vegetable scores (Fig. 1 and online supplementary material, Supplemental Table 1). The odds were lower also for scores in sugar-sweetened beverages (SSB), fibre-rich grains, fruits and berries, vegetable oil, fish, nuts and seeds and milk, both before and after adjustment for sociodemographic factors. On the contrary, compared with food-secure participants, those with severe food insecurity had 1·15-fold odds of having a higher (more optimal) score in red and processed meat. The odds did not differ between the food security levels in the scores for fat spreads and snacks.

## Discussion

Our main finding was that severe food insecurity was associated with overall lower diet quality than in those without food insecurity. More specifically, severe food insecurity was linked to less frequent consumption of vegetables, fruits and berries, fibre-rich grains, fish, vegetable oil and nuts and seeds and more frequent consumption of SSB. Since lower consumption of these food groups (apart from SSB) is all linked to various adverse health outcomes^(14)^, participants who experienced severe food insecurity may be predisposed to cumulation of risk factors for chronic diseases over time.

Our findings are mainly in line with studies from other countries that suggest lower diet quality in individuals who experience food insecurity in terms of both overall diet^(13,15–19)^ and individual food groups^(13,15–17)^. Regarding consumption of individual food groups, Lund *et al.*^(15)^ found similar results to ours among Danish adults (*n* 1877); food insecurity was associated with a lower intake of fruits, vegetables and fish, which were also the food groups in which we found the largest differences between severely food-insecure and food-secure participants. This could be because of the preference for ‘cheap energy’ as discussed later.

Interestingly, severe food insecurity was associated with less frequent consumption of red and processed meat than in the food-secure group. As limiting the consumption of red and processed meat is recommended^(31)^, in this regard, the diet of the food-insecure group could be viewed as healthier. Alternatively, the finding could be an indication of low energy intake, which we cannot rule out because we did not measure energy intake. A small number of studies in high-income countries have not found an association between food insecurity and energy intake^(16,20,37)^. We also do not know whether red and processed meat were replaced with less nutritious food items, in which case the infrequent intake would not indicate a healthier diet. Consequently, it is difficult to draw conclusions about the healthiness of the less frequent use of red and processed meat by the severely food-insecure participants. Nevertheless, one reason for less frequent consumption of red and processed meat could be the high price of some red meat products^(38,39)^ although the variation in the prices of red meat products is large^(40)^. Previous studies suggest that meat is also an established part of low-income households’ diets^(41,42)^ and that its consumption has not been associated with income^(40,42)^. However, the participants in those studies were not identified as food insecure and therefore may have still had more financial flexibility to make food choices based on preference – something that may not be possible for individuals experiencing food insecurity. A somewhat similar result to our high consumption of SSB by food-insecure individuals is that of a French study of a nationally representative population (*n* 2624)^(16)^. The researchers found that consumption of soft drinks was high in both those with food insecurity and those with the lowest income without food insecurity, compared with subjects with a higher income. An American study on dental students (*n* 286) also found higher sugar intake from SSB among those experiencing food insecurity than among food-secure individuals^(17)^. Although the criteria for high consumption differed between the French and American studies and our study, the direction of the association was similar.

It should be noted that to be categorised as severely food insecure in our study required skipping meals several times and/or going a whole day without eating at least once during the past month. Hence, it is logical that the consumption frequency is lower in many food categories, as severely food-insecure participants, by definition, ate less frequently than food-secure participants. It could, however, be speculated that people with severe food insecurity prefer cheaper sources of energy, such as refined grains and sugary drinks, instead of the foods they consume less frequently, including fruits, vegetables and fish. Earlier studies have demonstrated that foods of lower nutritional value, and lower-quality diets in general, cost less per calorie and tend to be favoured by groups of lower socio-economic status^(43)^. In a Belgian study, consumers who spent less money on food were more likely to fail to meet healthy dietary guidelines^(44)^. Food insecurity has also been linked to overweight and obesity, and a previous study suggested that obesity was a mediator between food insecurity and cardiometabolic diseases^(1)^.

The results of this study must be interpreted with certain limitations in mind. First, due to the cross-sectional study design, conclusions on causality cannot be made. We do not know the duration of food insecurity or possible changes in the diet, as the measures only consider the past month. The main limitations of food consumption assessment were the self-reporting of consumption frequency, with no measurement of portion sizes or energy intake. Those who experience food insecurity tend to consume smaller portion sizes^(45)^, but we did not consider this. In addition, we were unable to adjust the analysis for energy intake, which is a potential confounder. However, in the validation study of the HFII, the correlation coefficients between the HFII scores and nutrient intakes measured with food records did not change substantially when adjusted for energy intake^(30)^. In addition, as is common in health research, the participants, on average, had a higher socio-economic status than typical private sector service workers, suggesting that the results may not be generalisable to all private sector service workers.

One notable strength of the study was that the HFIAS tool for measuring food insecurity is validated in this population^(10)^. The measures for food intake and diet quality were not validated in this population, but the original FFQ has previously been validated in Finnish children^(29)^ and the original HFII in pregnant Finnish women^(30)^. Our HFII modifications included the important food group of red and processed meat, as well as nuts and seeds, thereby improving the HFII to better reflect current dietary recommendations. Also, the FFQ allowed us to examine food intake over a longer time period than, for example, food diaries or 24 h recalls, which only consider short periods.

Another strength is that we were able to investigate a typically hard-to-reach population – low-paid private sector service workers – who are usually underrepresented in studies. The representativeness of the current sample of the low-salary private sector worker population is described in more detail in Walsh *et al.*^(10)^, but it can be concluded that despite some limitations we were able to capture Finnish-speaking private service sector union non-student members reasonably well. However, because our research only focused on members of PAM, individuals from some of the most vulnerable groups who are less frequently members of trade unions are probably underrepresented, namely, young people, men, unemployed and those in part-time or fixed-term contracts^(33)^. In addition, because the questionnaires were not translated into other languages, the study population may have consisted of lesser diversity due to the high likelihood of missing non-Finnish-speaking members. According to PAM’s own data, 6·2 % of its members have foreign background^(26)^. Immigrant background has been identified as a risk factor for food insecurity^(46)^, and ethnicity has been found to moderate the association between food insecurity and diet quality^(18)^. Therefore, more inclusive data collection methods should be considered in the future.

Low intake of healthy foods, such as fruits, vegetables and fish, is more common in socio-economically disadvantaged groups^(36)^. Our findings suggest an even more concerning situation: clustering of suboptimal consumption across various food groups among those experiencing severe food insecurity. Our findings highlight the urgency of implementing effective actions to ensure equal access to a healthy diet. Potential actions include reforms in food taxation and the availability of affordable, nutritious meals in workplace restaurants. However, the primary focus should be on decreasing poverty among workers through sufficient salaries, fair employment contracts and robust social security to prevent food insecurity^(10)^.

Food insecurity is a relevant issue also due to elevated food costs. The cost of food in Finland increased by 16 % in March 2023 from March 2022^(47)^. It is reasonable to assume that rising costs will further drive people towards food insecurity and worsen the situation for those already affected, as could be the case for 65 % of the PAM members in the present sample. Our results suggest that private sector service workers are at increased risk of non-communicable diseases not only because of more prevalent food insecurity but also because of lower diet quality^(2)^. In addition, food insecurity and poor diet quality are associated with worse work ability and more frequent health care utilisation^(48–50)^. More research is warranted on the long-term implications and interconnections between food insecurity, diet quality, health and the societal impacts these may have.

## Supporting information

Joutsi et al. supplementary material Joutsi et al. supplementary material

## Supplementary material

For supplementary material accompanying this paper, visit https://doi.org/10.1017/S1368980024002386.

## References

[1] Te Vazquez J , Feng SN , Orr CJ et al. (2021) Food insecurity and cardiometabolic conditions: a review of recent research. Curr Nutr Rep 10, 243–254. 34152581 10.1007/s13668-021-00364-2 PMC8216092

[2] Seligman HK , Bindman AB , Vittinghoff E et al. (2007) Food insecurity is associated with diabetes mellitus: results from the national health examination and nutrition examination survey (NHANES) 1999–2002. J Gen Intern Med 22, 1018–1023. 17436030 10.1007/s11606-007-0192-6 PMC2583797

[3] Men F , Gundersen C , Urquia ML et al. (2020) Association between household food insecurity and mortality in Canada: a population-based retrospective cohort study. CMAJ 192, E53–60. 31959655 10.1503/cmaj.190385 PMC6970600

[4] Gundersen C (2016) Understanding food insecurity in the USA and Canada: potential insights for Europe. World Rev Nutr Diet 115, 54–60. 27198157 10.1159/000442072

[5] Borch A & Kjærnes U (2016) Food security and food insecurity in Europe: an analysis of the academic discourse (1975–2013). Appetite 103, 137–147. 27067740 10.1016/j.appet.2016.04.005

[6] Kötzsche M , Teuber R , Jordan I et al. (2023) Prevalence and predictors of food insecurity among university students – results from the Justus Liebig University Giessen, Germany. Prev Med Rep 36, 102526. 38116256 10.1016/j.pmedr.2023.102526 PMC10728436

[7] Campanera M , Gasull M & Gracia-Arnaiz M (2023) Food security as a social determinant of health. Health Hum Rights 25, 9–21. 37266309 PMC9973507

[8] Statistics (2019) World Food and Agriculture - Statistical Pocketbook 2019. Rome, Italy: FAO.

[9] Sarlio-Lähteenkorva S & Lahelma E (2001) Food insecurity is associated with past and present economic disadvantage and body mass index. J Nutr 131, 2880–2884. 11694612 10.1093/jn/131.11.2880

[10] Walsh HM , Nevalainen J , Saari T et al. (2022) Food insecurity among Finnish private service sector workers: validity, prevalence and determinants. Public Health Nutr 25, 829–840. 35067259 10.1017/S1368980022000209 PMC9993037

[11] Erkkola M , Walsh HM , Saari T et al. (2023) Private sector service workers’ well-being before and during the COVID-19 pandemic. Soc Sci Humanit Open 8, 100711.

[12] Ahtiainen L (2023) Palkansaajien Järjestäytyminen Vuonna 2021 (Organization of Employees into Trade Unions in 2021). Helsinki: Ministry of Economic Affairs and Employment.

[13] Hanson KL & Connor LM (2014) Food insecurity and dietary quality in US adults and children: a systematic review. Am J Clin Nutr 100, 684–692. 24944059 10.3945/ajcn.114.084525

[14] Afshin A , Sur PJ , Fay KA et al. (2019) Health effects of dietary risks in 195 countries, 1990–2017: a systematic analysis for the global burden of disease study 2017. Lancet 393, 1958–1972. 30954305 10.1016/S0140-6736(19)30041-8 PMC6899507

[15] Lund TB , Holm L , Tetens I et al. (2018) Food insecurity in Denmark-socio-demographic determinants and associations with eating- and health-related variables. Eur J Public Health 28, 283–288. 29020375 10.1093/eurpub/ckx121

[16] Bocquier A , Vieux F , Lioret S et al. (2015) Socio-economic characteristics, living conditions and diet quality are associated with food insecurity in France. Public Health Nutr 18, 2952–2961. 25563304 10.1017/S1368980014002912 PMC10271578

[17] Marshall TA , Laurence B , Qian F et al. (2023) Food insecurity is associated with lower diet quality among dental students. J Dent Educ 87, 1574–1584. 37537836 10.1002/jdd.13344

[18] Leung CW & Tester JM (2019) The association between food insecurity and diet quality varies by race/ethnicity: an analysis of national health and nutrition examination survey 2011–2014 results. J Acad Nutr Diet 119, 1676–1686. 30579633 10.1016/j.jand.2018.10.011 PMC6584552

[19] Keenan GS , Christiansen P & Hardman CA (2021) Household food insecurity, diet quality, and obesity: an explanatory model. Obesity (Silver Spring) 29, 143–149. 33135388 10.1002/oby.23033

[20] Shinwell J , Bateson M , Nettle D et al. (2022) Food insecurity and patterns of dietary intake in a sample of UK adults. Br J Nutr 128, 770–777. 34551836 10.1017/S0007114521003810 PMC9346616

[21] Darmon N & Drewnowski A (2008) Does social class predict diet quality?. Am J Clin Nutr 87, 1107–1117. 18469226 10.1093/ajcn/87.5.1107

[22] Prättälä R , Linnanmäki E & Vartiainen E (2008) Terveyserojen Kaventaminen Elintapoihin Vaikuttamalla, Liite 5 Teoksessa Kansallinen Terveyserojen Kaventamisen Toimintaohjelma 2008–2011. Helsinki: Sosiaali- Ja Terveysministeriö.

[23] Holmes B (2008) The influence of food security and other social and environmental factors on diet in the national low income diet and nutrition survey. Proc Nutr Soc 67, E88. 18768095 10.1017/S0029665108007209

[24] Álvares L & Amaral TF (2014) Food insecurity and associated factors in the Portuguese population. Food Nutr Bull 35, 395–402. 25639124 10.1177/156482651403500401

[25] Tarasuk V , Fafard St-Germain AA & Mitchell A (2019) Geographic and socio-demographic predictors of household food insecurity in Canada, 2011–12. BMC Public Health 19, 12. 30606152 10.1186/s12889-018-6344-2 PMC6318847

[26] PAM (n.d.) About PAM. Available from: https://www.pam.fi/en/about-pam.html (accessed April 2023).

[27] Coates J , Swindale A & Bilinsky P (2007) Household Food Insecurity Access Scale (HFIAS) for Measurement of Food Access: Indicator Guide: Version 3: (576842013-001). Washington, D.C.: American Psychological Association.

[28] FAO (2016) Methods for Estimating Comparable Rates of Food Insecurity Experienced by Adults Throughout the World. Rome: FAO.

[29] Korkalo L , Vepsäläinen H , Ray C et al. (2019) Parents’ reports of preschoolers’ diets: relative validity of a food frequency questionnaire and dietary patterns. Nutrients 11, 159. 30642103 10.3390/nu11010159 PMC6356196

[30] Meinilä J , Valkama A , Koivusalo SB et al. (2016) Healthy food intake index (HFII) – validity and reproducibility in a gestational-diabetes-risk population. BMC Public Health 16, 680. 27475905 10.1186/s12889-016-3303-7 PMC4967513

[31] Blomhoff R , Andersen R , Arnesen EK et al. (2023) Nordic Nutrition Recommendations 2023: Integrating Environmental Aspects. Copenhagen: Nordic Council of Ministers.

[32] National Nutrition Council (2014) Suomalaiset Ravitsemussuositukset – Terveyttä Ruoasta [Finnish Nutrition Recommendations – Health from Food]. Tampere, Finland: Juvenes Print.

[33] Jonker-Hoffrén P (2019) Goodbye centralised bargaining? The emergence of a new industrial bargaining regime. In Collective Bargaining in Europe: Towards an Endgame, pp. 197–216, [ T Müller , K Vandaele & J Waddington , editors]. Brussels: European Trade Union Institute.

[34] Grimaccia E & Naccarato A (2019) Food insecurity individual experience: a comparison of economic and social characteristics of the most vulnerable groups in the world. Soc Indic Res 143, 391–410.

[35] Konttinen H , Halmesvaara O , Fogelholm M et al. (2021) Sociodemographic differences in motives for food selection: results from the LoCard cross-sectional survey. Int J Behav Nutr Phys Act 18, 71. 34078396 10.1186/s12966-021-01139-2 PMC8173871

[36] Valsta LM , Tapanainen H , Kortetmäki T et al. (2022) Disparities in nutritional adequacy of diets between different socioeconomic groups of Finnish adults. Nutrients 14, 1347. 35405960 10.3390/nu14071347 PMC9002951

[37] Zizza CA , Duffy PA & Gerrior SA (2008) Food insecurity is not associated with lower energy intakes. Obesity 16, 1908–1913. 18535545 10.1038/oby.2008.288

[38] Maillot M , Darmon N , Darmon M et al. (2007) Nutrient-dense food groups have high energy costs: an econometric approach to nutrient profiling. J Nutr 137, 1815–1820. 17585036 10.1093/jn/137.7.1815

[39] Drewnowski A , Darmon N & Briend A (2004) Replacing fats and sweets with vegetables and fruits—a question of cost. Am J Public Health 94, 1555–1559. 15333314 10.2105/ajph.94.9.1555 PMC1448493

[40] Fogelholm M , Vepsäläinen H , Meinilä J et al. (2024) The dynamics in food selection stemming from price awareness and perceived income adequacy: a cross-sectional study using 1-year loyalty-card data. Am J Clin Nutr 119, 1346–1353. 38458401 10.1016/j.ajcnut.2024.03.003 PMC11130695

[41] Wiig K & Smith C (2009) The art of grocery shopping on a food stamp budget: factors influencing the food choices of low-income women as they try to make ends meet. Public Health Nutr 12, 1726–1734. 19068150 10.1017/S1368980008004102

[42] Inglis V , Ball K & Crawford D (2009) Does modifying the household food budget predict changes in the healthfulness of purchasing choices among low- and high-income women?. Appetite 52, 273–279. 19013206 10.1016/j.appet.2008.10.005

[43] Darmon N & Drewnowski A (2015) Contribution of food prices and diet cost to socioeconomic disparities in diet quality and health: a systematic review and analysis. Nutr Rev 73, 643–660. 26307238 10.1093/nutrit/nuv027 PMC4586446

[44] Vandevijvere S , Seck M , Pedroni C et al. (2021) Food cost and adherence to guidelines for healthy diets: evidence from Belgium. Eur J Clin Nutr 75, 1142–1151. 33239748 10.1038/s41430-020-00815-z

[45] Usfar AA , Fahmida U & Februhartanty J (2007) Household food security status measured by the US-household food security/hunger survey module (US-FSSM) is in line with coping strategy indicators found in urban and rural Indonesia. Asia Pac J Clin Nutr 16, 368–374. 17468096

[46] Maynard M , Dean J , Rodriguez PI et al. (2019) The experience of food insecurity among immigrants: a scoping review. J Int Migr Integr 20, 375–417.

[47] Tilastokeskus (2024) Statistics Finland. Statistics Finland; available at https://www.stat.fi/tup/suoluk/suoluk_hinnat_en.html#Consumer%20price%20index%202015 %20=%20100 %20by%20group%20of%20goods%20and%20services (accessed January 2024).

[48] Penttinen MA , Virtanen J , Laaksonen M et al. (2021) The association between healthy diet and burnout symptoms among Finnish municipal employees. Nutrients 13, 2393. 34371901 10.3390/nu13072393 PMC8308766

[49] Tarasuk V , Cheng J , de Oliveira C et al. (2015) Association between household food insecurity and annual health care costs. CMAJ 187, E429–36. 26261199 10.1503/cmaj.150234 PMC4592315

[50] Virtanen J , Penttinen MA , Laaksonen M et al. (2022) The relationship between dietary habits and work engagement among female Finnish municipal employees. Nutrients 14, 1267. 35334924 10.3390/nu14061267 PMC8949237

