# Supplementary material for: Does food insecurity compromise diet quality among Finnish private sector service workers?
Source: Public Health Nutr. 2024 Nov 22;27(1):e250. doi: 10.1017/S1368980024002386 (PMC11705014; doi:10.1017/S1368980024002386)
Supplement: Joutsi et al. supplementary material [file S1368980024002386sup001.docx]

Supplemental Table 1. Number of participants in the component score categories of the modified Healthy Food Intake Index (HFII) by food security status extremities (severely food insecure / food secure), and odds ratios and their 95% confidence intervals for severely food insecure to have higher food group score than food secure (ordinal regression). Study population: Finnish private sector service workers (n=4564-4579) in 2019.

|  |  | Severely food insecure |  | Food secure |  |  |  |  |
| --- | --- | --- | --- | --- | --- | --- | --- | --- |
|  |  | n | % | n | % | OR | 95 % CI | p |
| Spreads | |  |  |  |  |  |  |  |
|  | 2 | 387 | 17 | 399 | 18 |  |  |  |
|  | 1 | 881 | 38 | 829 | 36 |  |  |  |
|  | 0 | 1031 | 45 | 1052 | 46 |  |  |  |
|  | Without adjusting |  |  |  |  | 1.03 | [0.92, 1.14] | =0.65 |
|  | Adjusted* |  |  |  |  | 1.04 | [0.93, 1.17] | =0.50 |
| Milk |  |  |  |  |  |  |  |  |
|  | 2 | 726 | 31 | 822 | 36 |  |  |  |
|  | 1 | 1076 | 47 | 914 | 40 |  |  |  |
|  | 0 | 497 | 22 | 544 | 24 |  |  |  |
|  | Without adjusting |  |  |  |  | 0.93 | [0.84, 1.04] | =0.21 |
|  | Adjusted* |  |  |  |  | 0.89 | [0.80, 1.00] | =0.049 |
| Snacks | |  |  |  |  |  |  |  |
|  | 2 | 862 | 37 | 817 | 36 |  |  |  |
|  | 1 | 706 | 31 | 677 | 30 |  |  |  |
|  | 0 | 731 | 32 | 786 | 34 |  |  |  |
|  | Without adjusting |  |  |  |  | 1.10 | [0.99, 1.22] | =0.08 |
|  | Adjusted* |  |  |  |  | 1.06 | [0.95, 1.19] | =0.30 |
| Sugar-sweetened beverages | | |  |  |  |  |  |  |
|  | 1 | 1048 | 46 | 1385 | 61 |  |  |  |
|  | 0 | 1251 | 54 | 895 | 39 |  |  |  |
|  | Without adjusting |  |  |  |  | 0.54 | [0.48, 0.61] | <0.001 |
|  | Adjusted* |  |  |  |  | 0.67 | [0.59, 0.76] | <0.001 |
| Red and processed meat | |  |  |  |  |  |  |  |
|  | 2 | 608 | 26 | 525 | 23 |  |  |  |
|  | 1 | 383 | 17 | 382 | 17 |  |  |  |
|  | 0 | 1308 | 57 | 1373 | 60 |  |  |  |
|  | Without adjusting |  |  |  |  | 1.16 | [1.04, 1.30] | =0.009 |
|  | Adjusted* |  |  |  |  | 1.15 | [1.02, 1.29] | =0.025 |
| Fibre-rich grains | |  |  |  |  |  |  |  |
|  | 2 | 522 | 23 | 701 | 31 |  |  |  |
|  | 1 | 997 | 43 | 1036 | 45 |  |  |  |
|  | 0 | 780 | 34 | 543 | 24 |  |  |  |
|  | Without adjusting |  |  |  |  | 0.63 | [0.57, 0.71] | <0.001 |
|  | Adjusted* |  |  |  |  | 0.79 | [0.71, 0.89] | <0.001 |
| Vegetables | |  |  |  |  |  |  |  |
|  | 1 | 1785 | 78 | 1884 | 83 |  |  |  |
|  | 0 | 514 | 22 | 396 | 17 |  |  |  |
|  | Without adjusting |  |  |  |  | 0.50 | [0.45, 0.56] | <0.001 |
|  | Adjusted* |  |  |  |  | 0.54 | [0.49, 0.61] | <0.001 |
| Fruits and berries | |  |  |  |  |  |  |  |
|  | 1 | 1000 | 43 | 1365 | 60 |  |  |  |
|  | 0 | 1299 | 57 | 915 | 40 |  |  |  |
|  | Without adjusting |  |  |  |  | 0.52 | [0.46, 0.58] | <0.001 |
|  | Adjusted* |  |  |  |  | 0.61 | [0.54, 0.69] | <0.001 |
| Vegetable oil | |  |  |  |  |  |  |  |
|  | 1 | 1785 | 78 | 1884 | 83 |  |  |  |
|  | 0 | 514 | 22 | 396 | 17 |  |  |  |
|  | Without adjusting |  |  |  |  | 0.73 | [0.63, 0.84] | <0.001 |
|  | Adjusted* |  |  |  |  | 0.80 | [0.69, 0.93] | =0.005 |
| Fish | |  |  |  |  |  |  |  |
|  | 2 | 983 | 43 | 1327 | 58 |  |  |  |
|  | 0 | 1316 | 57 | 953 | 42 |  |  |  |
|  | Without adjusting |  |  |  |  | 0.54 | [0.48, 0.60] | <0.001 |
|  | Adjusted* |  |  |  |  | 0.65 | [0.57, 0.73] | <0.001 |
| Nuts and seeds | |  |  |  |  |  |  |  |
|  | 1 | 286 | 12 | 458 | 20 |  |  |  |
|  | 0 | 2013 | 88 | 1822 | 80 |  |  |  |
|  | Without adjusting |  |  |  |  | 0.56 | [0.48, 0.66] | <0.001 |
|  | Adjusted* |  |  |  |  | 0.66 | [0.55, 0.78] | <0.001 |

* Adjusted for age, sex, and education level.
